# Species diversity and insecticide resistance within the *Anopheles hyrcanus* group in Ubon Ratchathani Province, Thailand

**DOI:** 10.1186/s13071-020-04389-4

**Published:** 2020-10-17

**Authors:** Anchana Sumarnrote, Hans J. Overgaard, Vincent Corbel, Kanutcharee Thanispong, Theeraphap Chareonviriyaphap, Sylvie Manguin

**Affiliations:** 1 Department of Entomology, Faculty of Agriculture at Kamphaeng Saen, Kasetsart University, Kamphaeng Saen Campus, Nakhon Pathom, Thailand; 2 Maladies Infectieuses et Vecteurs, Ecologie, Génétique, Evolution et Contrôle (MIVEGEC), Institut de Recherche pour le Développement (IRD), University of Montpellier, Montpellier, France; 3 Faculty of Science and Technology, Norwegian University of Life Sciences, Ås, Norway; 4 Bureau of Vector-borne Disease, Department of Disease control, Ministry of Public Health, Nonthaburi, Thailand; 5 Department of Entomology, Faculty of Agriculture, Kasetsart University, Bangkok, Thailand; 6 HydroSciences Montpellier (HSM), Institut de Recherche pour le Développement (IRD), CNRS, Université Montpellier, Montpellier, France

**Keywords:** *Anopheles hyrcanus*, Malaria vectors, Species diversity, Insecticide resistance, Ubon Ratchathani Province, Thailand

## Abstract

**Background:**

Members of the *Anopheles hyrcanus* group have been incriminated as important malaria vectors. This study aims to identify the species and explore the insecticide susceptibility profile within the *Anopheles hyrcanus* group in Ubon Ratchathani Province, northeastern Thailand where increasing numbers of malaria cases were reported in 2014.

**Methods:**

Between 2013 and 2015, five rounds of mosquito collections were conducted using human landing and cattle bait techniques during both the rainy and dry seasons. *Anopheles* mosquitoes were morphologically identified and their insecticide susceptibility status was investigated. Synergist bioassays were carried out with *An. hyrcanus* (*s.l*.) due to their resistance to all insecticides. An ITS2-PCR assay was conducted to identify to species the Hyrcanus group specimens.

**Results:**

Out of 10,361 *Anopheles* females collected, representing 18 taxa in 2 subgenera, 71.8% were morphologically identified as belonging to the Hyrcanus Group (subgenus *Anopheles*), followed by *An. barbirostris* group (7.9%), *An. nivipes* (6.5%), *An. philippinensis* (5.9%) and the other 14 *Anopheles* species. Specimens of the Hyrcanus Group were more prevalent during the rainy season and were found to be highly zoophilic. *Anopheles hyrcanus* (*s.l*.) was active throughout the night, with an early peak of activity between 18:00 h and 21:00 h. ITS2-PCR assay conducted on 603 DNA samples from specimens within the Hyrcanus Group showed the presence of five sisters species. *Anopheles peditaeniatus* was the most abundant species (90.5%, *n* = 546), followed by *An. nitidus* (4.5%, *n* = 27), *An. nigerrimus* (4.3%, *n* = 26), *An. argyropus* (0.5%, *n* = 3), and *An. sinensis* (0.2%, *n* = 1). All *An. hyrcanus* (*s.l*.) specimens that were found resistant to insecticides (deltamethrin 0.05%, permethrin 0.75% and DDT 4% and synergist tests) belonged to *An. peditaeniatus*. The degree of resistance in *An. peditaeniatus* to each of these three insecticides was approximately 50%. Addition of PBO (Piperonyl butoxide), but not DEF (S.S.S-tributyl phosphotritioate), seemed to restore susceptibility, indicating a potential role of oxidases as a detoxifying enzyme resistance mechanism.

**Conclusions:**

A better understanding of mosquito diversity related to host preference, biting activity and insecticide resistance status will facilitate the implementation of locally adapted vector control strategies.
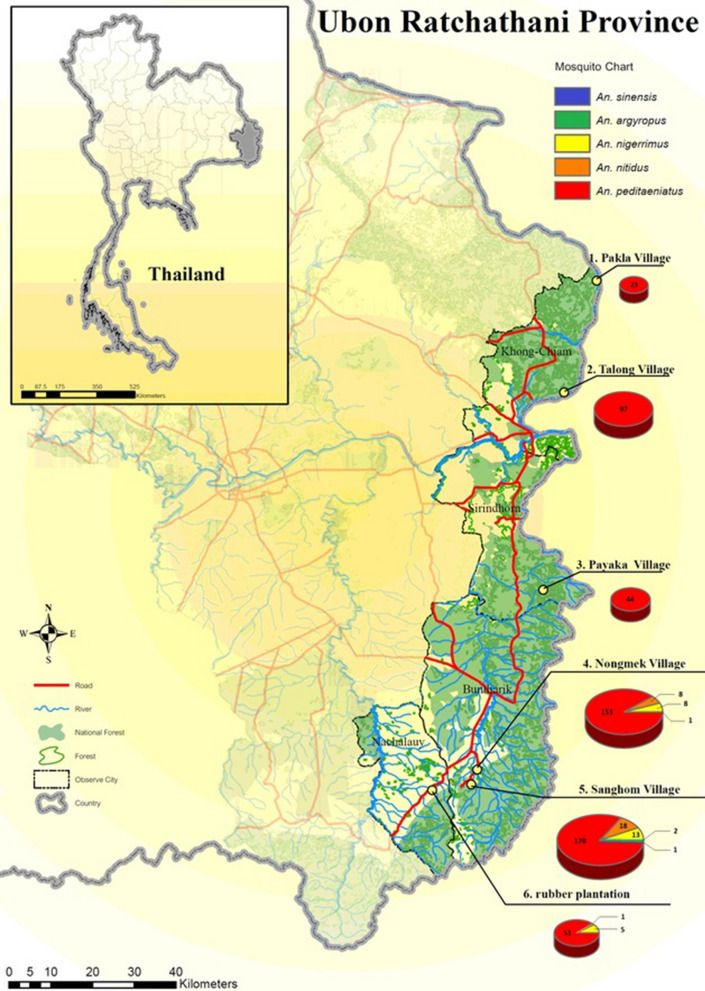

## Background

In the southern Palearctic and Oriental regions, the Hyrcanus Group of mosquitoes is a complex species assemblage belonging to the Myzorhynchus Series of the subgenus *Anopheles* (Diptera: Culicidae), which comprises 26 closely related species [1, 2]. Several species of this group have previously been identified as active malaria vectors transmitting *Plasmodium vivax*, as well as lymphatic filariasis caused by *Brugia malayi* and *Wuchereria bancrofti* in many countries of South, Southeast and East Asian regions [3, 4]. Within the Hyrcanus Group, *Anopheles sinensis* is considered the most efficient vector of these human parasitic agents, especially *P. vivax*, in China and Korea, and *P. malariae* in Vietnam [4–11]. *Anopheles peditaeniatus* has been reported as a secondary vector of Japanese encephalitis virus in China and India [12–14]. In southern Vietnam, *An. nimpe* transmits both *P. falciparum* and *P. vivax* [6, 15, 16]. *Anopheles nigerrimus* was incriminated as a suspected vector of *P. falciparum* and *P. vivax* in Bangladesh [17] and as a secondary or incidental vector of *W. bancrofti* in Asian regions [3], although in China this species is of primary importance in the transmission of *W. bancrofti* [18] and the arthropod roundworm *Romanomermis jingdeensis* [19]. In central China, *An. lesteri* (syn. *An. anthropophagus)*, which was considered as the primary malaria vector [20], is disappearing for the benefit of *An. sinensis*, which is the predominant species in southwestern China [10]. In addition, the species of the Hyrcanus Group are also considered as economic pests of cattle due to their vicious biting behavior and ability to transmit cervid filariae of the genus *Setaria* [21, 22].

In Thailand, at least eight species of the Hyrcanus Group have been reported so far. These include: three species of the Lesteri Subgroup (*An. crawfordi*, *An. paraliae* and *An. peditaeniatus*), three species of the Nigerrimus Subgroup (*An. nigerrimus*, *An. nitidus* and *An. pursati*) and two unassociated species (*An. argyropus* and *An. sinensis*) [23, 24]. Seven of these species are widely distributed throughout the country, while *An. paraliae* is restricted to coastal areas of peninsular and southeastern Thailand [24]. *Anopheles argyropus*, *An. nigerrimus*, *An. peditaeniatus*, and *An. sinensis* are the most abundant of the Hyrcanus species and are widely distributed in Thailand where they are found in valleys and mountainous areas [24]. *Anopheles nitidus* and *An. crawfordi* are frequent in forested areas. *Anopheles paraliae* larvae are normally developing in shaded semi- to permanent brackish water but not in rice fields, contrasting with immature stages of most species of the Hyrcanus Group, which are usually observed in rice fields, marshy and swampy areas, ponds, and other similar habitats with emergent vegetation [24]. Among the eight *An. hyrcanus* (*s.l*.) species recorded in Thailand, *An. sinensis*, *An. nigerrimus* and *An. peditaeniatus* are suspected as vectors of *P. vivax* [22, 25, 26]. Rattanarithikul et al. [26] have already detected the circumsporozoite antigen of *P. vivax* in *An. hyrcanus* (*s.l*.) specimens. Moreover, experimental infections of the Thai *An. hyrcanus* (*s.l*.) species (*An. argyropus*, *An. crawfordi*, *An. nigerrimus*, *An. peditaeniatus* and *An. pursati*) with the nocturnally subperiodic *B. malayi* filaria have been successfully conducted in the past, thus showing the vectorial potential of these mosquitoes for the transmission of this parasite [27].

Adult females within the Hyrcanus Group stand out from other *Anopheles* groups by the presence of basal pale scales on their antennae (usually four scales) and a tuft of dark scales on each side of the clypeus. In contrast, adult females of the Hyrcanus Group are difficult to distinguish due to their overlapping morphological characters, particularly when they occur in sympatry, thus leading to misidentification. In the field, Paredes-Esquivel et al. [28] reported frequent misidentification of *An. hyrcanus* (*s.l*.) specimens with members of the *An. barbirostris* group, which also belongs to the Myzorhynchus Series. Mosquitoes of the two groups share some similar morphological characteristics such as a humeral crossvein composed of a remigium patch with dark scales and narrow apical pale bands on the midtarsi, comparable wing patterns, narrow apical fringe spot on the wings, narrow tarsal bands, and highly variable hindtarsal banding patterns [23]. These characteristics, as well as loss or damage of mosquito samples during field activities, are the leading causes of misidentification that may skew data on the current distribution of *Anopheles* vectors across the country and ultimately reduce the benefits of vector control approaches. Accurate identification of *Anopheles* mosquitoes is therefore needed mainly because only a few species play an important role in malaria transmission and targeting the specific species is crucial for implementation of effective vector control measures.

The second internal transcribed spacer (ITS2) of nuclear ribosomal DNA and the cytochrome *c* oxidase subunits 1 and 2 (*cox*1 and *cox*2) of the mitochondrial DNA have been widely used to distinguish species of the Hyrcanus Group [7, 23, 25, 29]. Due to its high inter- and intra-specific variability, studies on the polymorphism of the ITS2 region have been helpful for the development of polymerase chain reaction (PCR) DNA-based assays in order to specifically identify cryptic and isomorphic species [30, 31]. The ITS2 region is relatively short in length (generally less than 1 kb) and highly conserved, which are suitable criteria for the design of universal primers allowing its fast amplification and sequencing [32]. Based on ITS2 polymorphisms, Hempolchom et al. [23] have designed specific primer sequences, which were successfully used in a single-round multiplex PCR for the identification of *Anopheles* species within the Hyrcanus Group.

In 2014, a malaria outbreak occurred in Ubon Ratchathani Province, located in the northeast of Thailand along the Cambodia and Lao PDR borders [33]. The number of malaria cases increased from 1130 in 2013 to 7708 cases in 2014, a seven-fold increment accounting for 26% of the total number of reported malaria cases in Thailand that year. Regarding the malaria transmission data in Thailand, only few entomological surveys have been conducted in the eastern part of the country. Consequently, little is known concerning the current distribution of *Anopheles* vectors in this malaria endemic area [34].

In addition, insecticide resistance in malaria vectors threatens malaria control efforts, which generates a growing concern in many countries. Insecticide resistance in some *Anopheles* mosquito have been previously reported in the Greater Mekong Subregion (GMS) countries. Van Bortel et al. [35] reported resistance to lambda-cyhalothrin and suspected resistance to alpha-cypermethrin in *An. dirus* (*sensu stricto*) in Vietnam. *Anopheles epiroticus* was reported to be resistant to all pyrethroid insecticides in the Mekong delta, while suspected resistance to DDT was found in Ho Chi Minh City [5]. *Anopheles minimus* (*sensu lato*) populations was found resistant to pyrethroids in northern Vietnam and to DDT in western Cambodia and northern Vietnam [35]. Resistance to DDT and pyrethroids was found in *Anopheles vagus* from Vietnam and Cambodia. In the northern part of Thailand, DDT resistance was detected in *An. dirus* (*s.l*.), *An. minimus* (*s.l*.) and *An. maculatus*. Resistance to permethrin was found in a population of *An. minimus* (*s.l*.) from northern Thailand [36]. Between 2013 and 2015, Sumarnrote et al. [37] conducted entomological surveys in order to assess species diversity and insecticide resistance to *Anopheles* collected in six sites along the Thai-Lao border. Our findings showed that *An. hyrcanus* group was the most represented taxon with a total of 7442 specimens corresponding to 72.5% of the total *Anopheles* population. Also, insecticide susceptibility assays were performed and we observed resistance to insecticides in most specimens collected (45 to 87% mortality to deltamethrin 0.05%, permethrin 0.75% and DDT 4%). It was relevant to address whether resistance occurred in potential malaria vectors. In order to complement the information on malaria transmission in potential endemic areas at high risk of outbreaks in Thailand, this present study aimed at identifying members of the Hyrcanus Group to species-level and explore the insecticide susceptibility profiles of *An. hyrcanus* (*s.l*.) mosquitoes in six locations across the Ubon Ratchathani Province.

## Methods

### Study sites

**Fig. 1:**
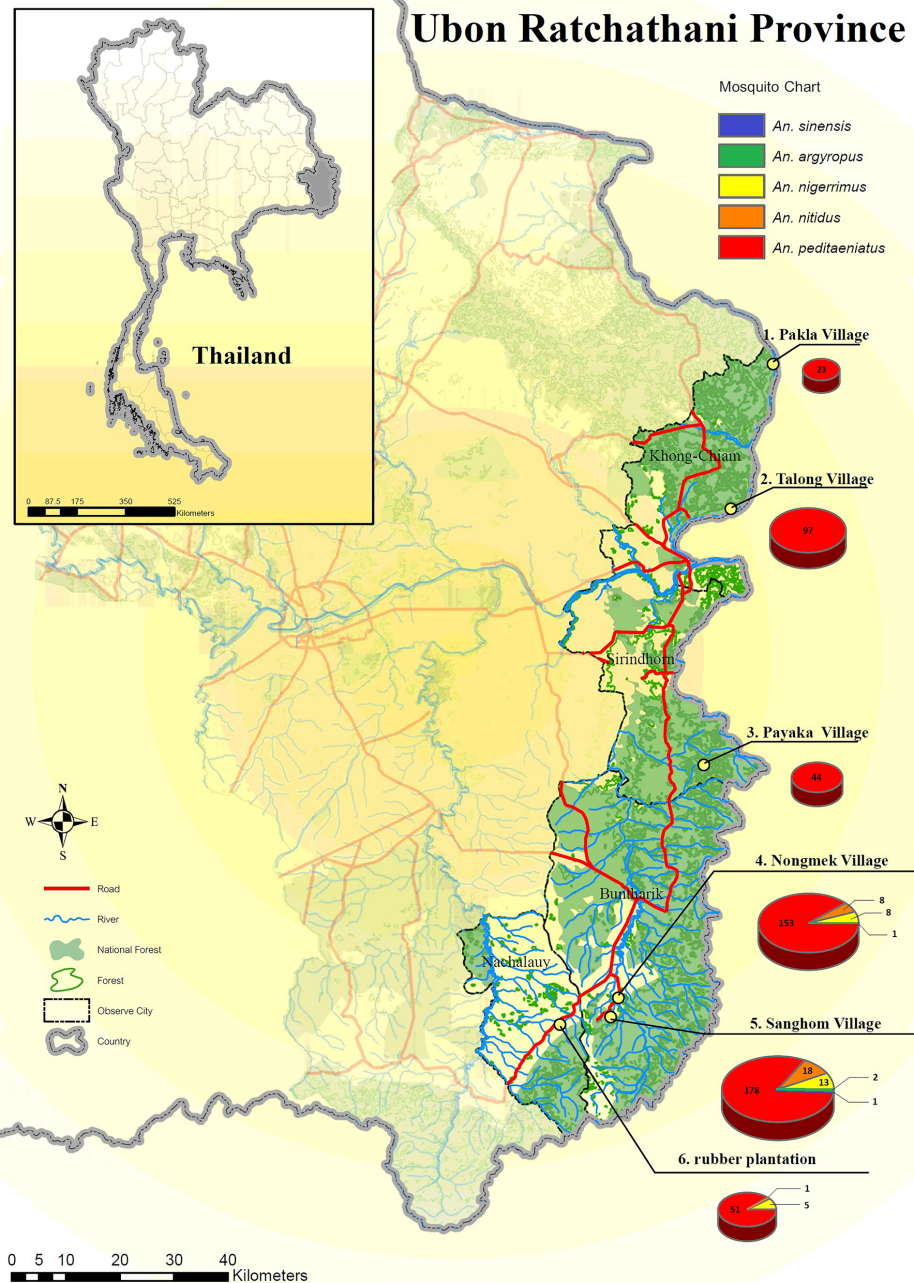
Collection sites located along on the Thailand-Laos border: 1, Pakla Village (Khong Chiam District); 2, Talong Village (Khong Chiam District); 3, Payaka Village (Sirindhorn District); 4, Nongmek Village (Buntharik District); 5, Sanghom Village (Buntharik District); and 6, Rubber plantation in Nachaluay District. The pie charts are proportional to the number of specimens collected and the number of species in the *An. hyrcanus* group identified by PCR

Mosquito collections were carried out in six sites (5 villages and 1 rubber plantation) located in four districts along the Thai-Lao border in Ubon Ratchathani Province (Fig. 1). The six surveyed sites included Pakla (15°38′46.0″N, 105°37′59.1″E) and Talong (15°24′20.4″N, 105°33′46.7″E) villages located in the Khong Chiam district, Nongmek (14°35′37.1″N, 105°22′33.5″E) and Sanghom (14°33′42.4″N, 105°21′47.5″E) villages in the Buntharik district, Payaka (14°58′49.8″N, 105°31′04.8″E) village in the Sirindhom district and the rubber plantation (14°32′57.2″N, 105°16′44.4″E) in the Nachaluay district. The selection of surveyed sites was based on malaria incidence data in the province between 2012 and 2015 [33]. During this 3 year-period, a total of 128,293 malaria cases were reported in Thailand with 14,079 (11%) cases recorded in the Ubon Ratchathani Province. Of these 14,079 cases, the Buntharik district had 6116 cases, followed by Nachalauy (4251 cases) and Nam Yuen (1593 cases), Si Mueang Mai (576 cases), Sirindhorn (357 cases), Det Udom (288 cases), and Khong Chiam (274 cases) [33].

### Mosquito collections and species identification

In each site, mosquito collections were conducted between September 2013 and September 2015 as described by Marasri et al. [38] and Sumarnrote et al. [37]. Collection periods are shown in Table 1. In brief, mosquitoes were collected during both dry (March) and rainy seasons (from September to October) over a two-year period using the human-landing catch (HLC) technique and cow bait collections (CBC) to determine the vector abundance and composition [39]. A separate collection for the susceptibility test along with the synergist test was conducted during the rainy season (September 2015). For every round of collection, four households and one cow fence were used respectively for HLCs (both indoor and outdoor) and CBC. The same collection points (households and cow fences) were maintained during the entire period of collection. Mosquitoes were individually placed in glass tubes and kept for morphological identification [24] and subsequent bioassays [40]. Then, specimens were preserved in microtubes at − 20 °C and brought back to the laboratory. *Anopheles* mosquitoes belonging to the Hyrcanus Group were sent to the UMR-HSM Laboratory at the Institut de Recherche pour le Développement, Montpellier, France, for molecular species identification using allele-specific multiplex PCR assays [23].

**Table 1:** Seasonal abundance of the Hyrcanus Group in the six locations in Ubon Ratchathani Province, Thailand

Collection location	Sub-district	District	Collection periods					
			Survey 1	Survey 2	Survey 3	Survey 4	Survey 5	Total
			Rainy	Dry	Rainy	Dry	Rainy	
			Sep-Oct 2013	Mar-2014	Sep-Oct 2014	Mar-2015	Sep-2015	
Pakla Village	Na Pho Klang	Khong Chiam	20	0	131	0	13	164
Talong Village	Huai Pai	Khong Chiam	187	0	568	0	–	755
Payaka Village	Non Ko	Sirindhorn	77	0	–	–	–	77
Nongmek Village	Huai Kha	Buntharik	563	13	779	3	–^a^	1358
Sanghom Village	Huai Kha	Buntharik	2124	14	1932	11	–^a^	4081
Rubber plantation	Nachaluay	Nachaluay	–	–	1001	6	–	1007
Total per survey^b^			2971	27	4411	20	13	7442

^a^Separate collections of Hyrcanus Group used for insecticide susceptibility test (not part of the regular collections)

^b^The total number of specimens collected during the rainy (surveys 1, 3, 5) and dry (surveys 2, 4) seasons were 7395 (99.4%) and 47 (0.6%), respectively

### Insecticide susceptibility of *An. hyrcanus* (*s.l*.) mosquitoes

According to our previous published study [37], susceptibility status to deltamethrin (0.05%), permethrin (0.75%) and DDT (4%) was investigated following WHO guidelines. Bioassays with the synergists PBO 4% and DEF 0.25% were carried out in *An. hyrcanus* (*s.l*.) to address the role of detoxifying enzymes in insecticide resistance. Knock-down resistance (*kdr*) was carried out to detect a fragment of the voltage-gated sodium channel gene substitutions at position 1014 in resistant mosquitoes.

### Extraction of mosquito genomic DNA

Genomic DNA was individually extracted from adult female *An. hyrcanus* (*s.l*.) using the whole insect according to previous protocols [41, 42]. Specifically, individual mosquitoes were placed into a DNA extraction tube and homogenized with 50 ml of extraction buffer containing 0.2 M sucrose, 0.1M Tris-HCl at pH 8.0, 50 mM EDTA (pH 8) and 0.5% SDS. Thereafter samples were incubated at 65 °C for 30 min. A volume of 11 μl of 5 mM KOAc (pH 9.0) was added in each tube and placed on ice for 30 min. After centrifugation for 20 min at 12,000× *rpm*, the supernatant was transferred into a clean tube. A volume of 100 μl of absolute ethanol was added and the samples were placed at 4 °C for 10 min. Samples were centrifuged at 12,000× *rpm* at 4 °C for 20 min and the supernatant discarded. The pellet was cleaned with 150 μl of 70% ethanol and centrifuged at 12,000× *rpm* for 5 min at 4 °C. The supernatant was discarded and the previous step was repeated with absolute ethanol and centrifugation at 12,000× *rpm* for 5 min at 4 °C. The resultant pellet was dried at room temperature for 30 min before being re-suspended in 100 μl of DNase/RNase-Free Distilled Water and stored at − 20 °C for further analyses.

### Molecular identification of *An. hyrcanus* sibling species by allele-specific PCR

The extracted DNA was used for species molecular identification using an allele-specific multiplex PCR assay (AS-PCR) examining the ITS2 region of the rDNA. The universal ITS2 forward primer (ITS2A) and eight species-specific reverse primers were used to selectively amplify genomic DNA of *An. hyrcanus* sibling species as described by Hempolchom et al. [23]. The PCR reaction was performed in a total volume of 20 μl, composed of 5× Tfi PCR reaction buffer (Invitrogen, Villebon-sur-Yvette, France), 50 mM MgCl_2_ (Invitrogen), 10 mM dNTPs (mix), and Tfi DNA polymerase (Taq) (5U/μl; Invitrogen). Thermal conditions were as follows, an initial denaturation temperature of 94 °C for 1 min, 30 cycles of 94 °C for 30 s, 55 °C for 30 s for the annealing temperature, an extension phase at 72 °C for 1 min and a final extension step at 72 °C for 5 min.

The amplified PCR products were subjected to electrophoresis in 2% agarose gels stained with GelRed (Biotium Inc, Fremont, CA, USA). The expected band sizes of the PCR amplicons for the different species are described by Hempolchom et al. [23]. Moreover, 20 PCR amplicons of different species were sent for sequencing and assessed by Genewiz (Paris, France) using the ITS2 universal primer (5′-TGT GAA CTG CAG GAC ACA T-3′) to confirm species identifications. Each sequence was checked and cleaned manually using the BioEdit software version 7.1.9 [﻿^43^].

### Data analysis

Percentage of zoophilic and anthropophilic behavior in *An. hyrcanus* group was assessed by the abundance of mosquito samples collected from CBC and HLC, respectively. The difference in the mean number of specimens of the *An. hyrcanus* group captured during the rainy and dry seasons was analyzed by non-parametric Mann-Whitney U-test and statistical significance was designated at 0.05% (*P*-value < 0.05). Statistical analyses were performed using the Statistical Package for Social Science (SPSS) version 24.0 (SPSS Inc., Chicago, IL, USA).

## Results

**Fig. 2:**
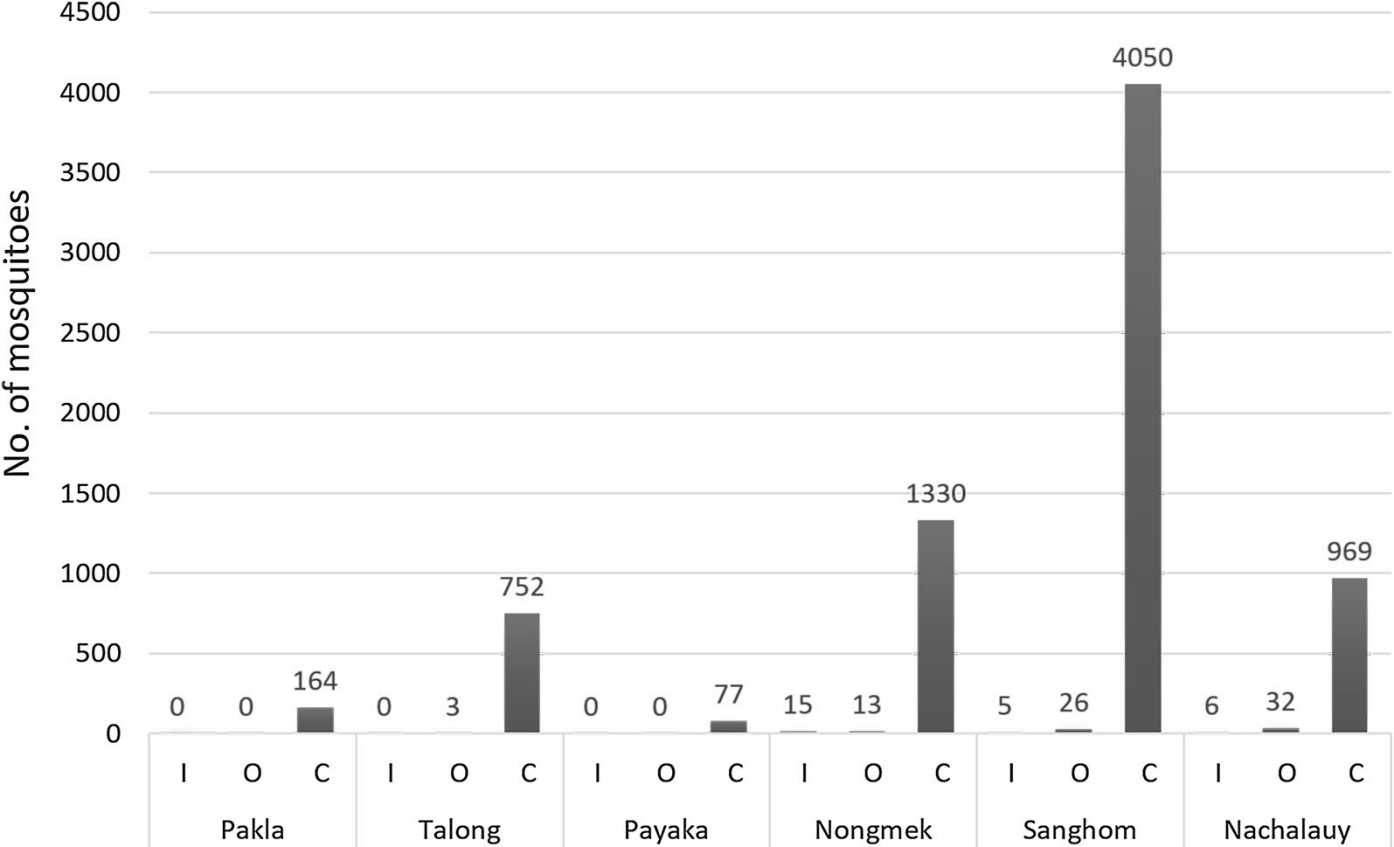
The number of specimens of the *An. hyrcanus* group collected on human (indoors and outdoors) and cattle bait. *Abbreviations*: I, indoors, O; outdoors, C; cow bait

**Fig. 3:**
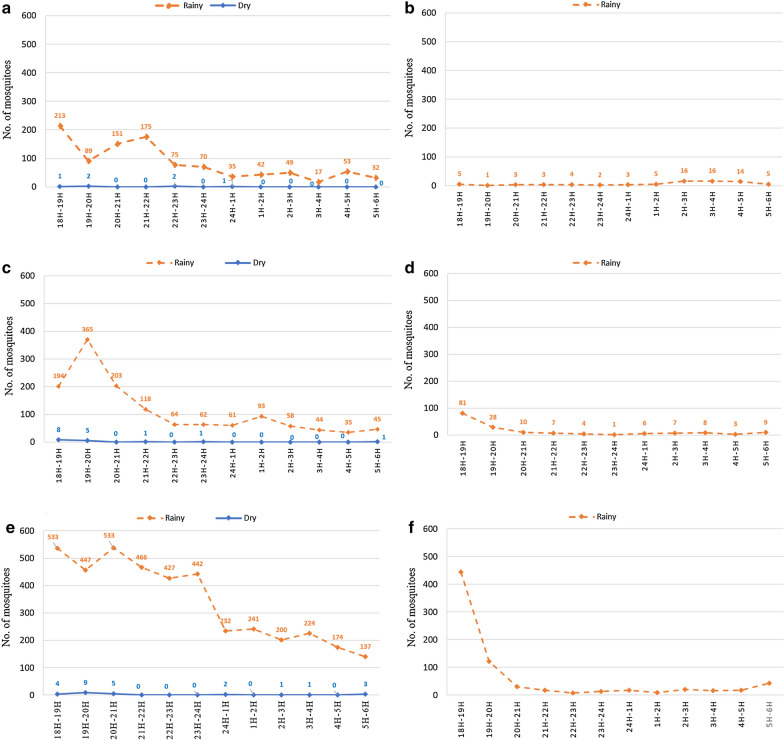
The number of mosquito specimens in the *An. hyrcanus* group collected per hour from each collection sites on cattle and human bait during the rainy and dry seasons in Ubon Ratchathani Province, Thailand. **a** Pakla. **b** Talong. **c** Nongmek. **d** Sanghom. **e** Payaka. **f** Rubber plantation in Nachaluay District

Overall, 10,361 females *Anopheles* mosquitoes were caught in the six villages over a 2-year (from September 2013 to September 2015) period of collection [38]. Of these, 7,442 specimens were morphologically identified as belonging to the *An. hyrcanus* group, thus corresponding to 71.8% of the total *Anopheles* fauna collected. Specimens of the *An. hyrcanus* group showed a zoophilic biting behavior with 98.7% collected on cow bait. Numbers of *An. hyrcanus* group collected on human and cattle bait from each site are presented in Fig. 2. The abundance of the Hyrcanus specimens with a brief description of the surrounding environment is presented in Additional file 1: Table S1. *Anopheles hyrcanus* (*s.l*.) was significantly (*P* < 0.001) more abundant during the rainy season (99.4%, 7395/7442) than during the dry season with only 47 specimens (0.6%). No *An. hyrcanus* was collected in Pakla, Talong and Payaka villages during the dry seasons (March 2014 and March 2015), whereas only a few specimens were collected in Nongmek (*n* =16) and Sanghom (*n* = 25) villages, and in the rubber plantation (*n* = 6) of the Nachaluay District (Table 1). The biting activity of the *An. hyrcanus* group was highest in the early evening during 18:00–21:00 h, and gradually declined thereafter (Fig. 3).

### Insecticide susceptibility tests and molecular species identification

Following insecticide susceptibility tests from our previous published study [37], a total of 603 specimens of the *An. hyrcanus* group representing different insecticide susceptibility status were selected and identified to species by a PCR-based assay. Samples consisted of mosquitoes that were either resistant (*n* = 230) or susceptible (*n* = 264) to various insecticides (Table 2).

**Table 2:** Number of specimens selected from the *An. hyrcanus* group mosquitoes collected in the study sites that were used for PCR-based species identification

Villages	Insecticide	Survey 1 Rainy		Survey 2 Dry		Survey 3 Rainy		Survey 4 Dry		Survey 5 Rainy		Total
		Alive	Dead	Alive	Dead	Alive	Dead	Alive	Dead	Alive	Dead	
Pakla	No test	10	–	–	–	–	–	–	–	2	–	12
	Deltamethrin 0.05%	–	–	–	–	–	–	–	–	5	6	11
	Permethrin 0.75%	–	–	–	–	–	–	–	–	–	–	0
	DDT 4%	–	–	–	–	–	–	–	–	–	–	0
	Control	–	–	–	–	–	–	–	–	–	–	0
Talong	Deltamethrin 0.05%	5	8	–	–	7	8	–	–			28
	Permethrin 0.75%	6	8	–	–	8	8	–	–			30
	DDT 4%	7	8	–	–	8	8	–	–			31
	Control	–	–	–	–	8	–	–	–			8
Payaka	Deltamethrin 0.05%	4	8	–	–							12
	Permethrin 0.75%	7	8	–	–							15
	DDT 4%	4	8	–	–							12
	Control	5	–	–	–							5
Nongmek	No test	–	–	6	–	–	–	2	–	–	–	8
	Deltamethrin 0.05%	8	5	–	–	8	8	–	–	8	8	45
	Permethrin 0.75%	10	8	–	–	8	8	–	–	–	–	34
	DDT 4%	9	8	–	–	8	8	–	–	–	–	33
	Control	10	–	–	–	7	–	–	–	8	–	25
	PBO+deltamethrin 0.05%	–	–	–	–	–	–	–	–	3	6	9
	DEF+deltamethrin 0.05%	–	–	–	–	–	–	–	–	8	8	16
Sanghom	No test	–	–	8	–	–	–	8	–	–	–	16
	Deltamethrin 0.05%	8	8	–	–	6	8	–	–	9	8	47
	Permethrin 0.75%	8	8	–	–	7	8	–	–	8	8	47
	DDT 4%	8	8	–	–	7	8	–	–	–	–	31
	Control	8	–	–	–	8	–	–	–	8	–	24
	PBO + deltamethrin 0.05%	–	–	–	–	–	–	–	–	–	8	8
	DEF + deltamethrin 0.05%	–	–	–	–	–	–	–	–	7	8	15
	PBO + permethrin 0.75%	–	–	–	–	–	–	–	–	–	8	8
	DEF + permethrin 0.75%	–	–	–	–	–	–	–	–	8	8	16
Nachaluay	No test	–	–	–	–	–	–	3	–	–	–	3
	Deltamethrin 0.05%					7	7	–	–			14
	Permethrin 0.75%					8	8	–	–			16
	DDT 4%					8	8	–	–			16
	Control					8	–	–	–			8
Total		117	93	14	0	121	95	13	0	74	76	603

*Notes*: Blank cells indicate that no mosquito collections were done during this period in this site

The PCR identification of 603 randomly selected samples identified as *An. hyrcanus* (*s.l*.) revealed the presence of five sibling species. *Anopheles peditaeniatus* was predominant (90.5%, 546/603) followed by *An. nitidus* (*n* = 27, 4.5%), *An. nigerrimus* (*n* = 26, 4.3%), *An. argyropus* (*n* = 3, 0.5%), and *An. sinensis* (*n* = 1, 0.2%). The highest abundance and species diversity were found in the village of Sanghom where all five *Anopheles* species were recorded (Table 3). In Pakla, Talong and Payaka villages, only *An. peditaeniatus* was found, while *An. peditaeniatus*, *An. nigerrimus*, *An. nitidus* in addition to *An. argyropus* were respectively recorded in Nachaluay and Nongmek villages (Table 3).

**Table 3:** Distribution of the *Anopheles hyrcanus* sibling species per study site based on PCR identification

Study site	Total number of mosquitoes collected	Number of *An. hyrcanus* (*s.l*.) mosquitoes identified by PCR				
		*An. peditaeniatus*	*An. argyropus*	*An. nigerrimus*	*An. nitidus*	*An. sinensis*
Pakla Village	164	23	–	–	–	–
Talong Village	755	97	–	–	–	–
Payaka Village	77	44	–	–	–	–
Nongmek Village	1358	153	1	8	8	–
Sanghom Village	4081	178	2	13	18	1
Rubber plantation (Nachaluay)	1007	51	–	5	1	–
Total	7442	546	3	26	27	1

Metabolic resistance mechanism in *Anopheles hyrcanus* (*s.l*.) was assessed by using synergists. In our previous publication, pre-exposure of *An. hyrcanus* (*s.l*.) to PBO 4% caused a significant increase of approximately 50% in mortality, whereas a slight increase of pyrethroid mortality was found in presence of DEF 0.25% [37]. Out of 564 specimens selected from bioassay tested with different insecticides as well as from the synergist tests, 230 resistant specimens were all identified as *An. peditaeniatus* by molecular assay (49.3%, 230 resistant out of 467 tested), while susceptible specimens were identified as *An. peditaeniatus* (*n* = 237), *An. nigerrimus* (*n* = 16) and *An. nitidus* (*n* = 9), *An. argyropus* (*n* = 1) and *An. sinensis* (*n* = 1) (Table 4).

**Table 4:** Species identification of *An. hyrcanus* group selected from bioassay tests by allele-specific PCR

Insecticide	Resistance status	Number of specimens					Total
		*An. peditaeniatus*	*An. nigerrimus*	*An. nitidus*	*An. argyropus*	*An. sinensis*	
Deltamethrin 0.05%	Susceptible	75	5	2	–	–	82
	Resistant	75	–	–	–	–	75
Permethrin 0.75%	Susceptible	64	5	3	–	–	72
	Resistant	70	–	–	–	–	70
DDT 4%	Susceptible	59	3	2	–	–	64
	Resistant	59	–	–	–	–	59
Piperonyl butoxide (PBO) 4% + deltamethrin 0.05%	Susceptible	12	2	–	–	–	14
	Resistant	3	–	–	–	–	3
Piperonyl butoxide (PBO) 4% + permethrin 0.75%	Susceptible	6	–	–	1	1	8
	Resistant	–	–	–	–	–	0
S.S.S-tributyl phosphotritioate (DEF) 0.25% + deltamethrin 0.05%	Susceptible	13	1	2	–	–	16
	Resistant	15	–	–	–	–	15
S.S.S-tributyl phosphotritioate (DEF) 0.25% + permethrin 0.75%	Susceptible	8	–	–	–	–	8
	Resistant	8	–	–	–	–	8
Control		66	2	2	–	–	70
Total		533	18	11	1	1	564
Resistant		230 (49.3%)	0	0	0	0	230
Susceptible		237 (50.7%)	16	9	1	1	264

## Discussion

Knowledge of the density and species composition of mosquito vectors is crucial for determining the distribution and potential risk of vector-borne diseases transmission. Previous findings showed that *An. hyrcanus* group is predominant along the Thai-Lao border of Ubon Ratchathani Province, accounting for 71.8% of all *Anopheles* species collected [38].

In this study, among the five species of the Hyrcanus Group identified, *An. peditaeniatus* was most abundant (90.5%, *n* = 546). This species is widely distributed in Thailand [24]. Gingrich et al. [44] reported that *An. peditaeniatus* was primarily caught during and after the rainy season and the biting peak was usually before midnight. The detection of the *Plasmodium* circumsprozoite antigen in *An. peditaeniatus* salivary glands using the enzyme-linked immunoassay (ELISA) raised up the potential implication of this species in malaria transmission across this region [44]. In Indonesia, *An. peditaeniatus* was found positive for *P. falciparum* circumsporozoite proteins (CSP) by testing heads or thoraces using ELISA [45]. St Laurent et al. [46] reported that one specimen of *An. peditaeniatus* collected in Cambodia was infected with *P. falciparum* using a nested PCR assay. Moreover, an *An. peditaeniatus*/*Brugia malayi* experimental model showed high susceptibility rate (ranging from 70 to 100%) of the vector to this microfilaria [27]. In addition, *An. peditaeniatus* is considered as a secondary vector of Japanese encephalitis virus in China and India [12, 14]. Although *An. peditaeniatus* is abundant and widely distributed throughout Thailand, its vectorial role remains uncertain and needs to be elucidated. Especially important for future studies will be to apply molecular assays to identify this species, as well as the other members of the Hyrcanus Group for precisely determining the role and behavior of each encountered member.

Host-seeking and biting preferences are other important indicators for assessing the role of *Anopheles* mosquitoes in malaria transmission. Our results showed that *An. hyrcanus* (*s.l*.) populations, specifically *An. peditaeniatus* (90.5%) have a high preference for cattle compared to humans, and therefore can be considered as highly zoophilic. However, morphological characters had solely been used for species identification, thus possible misidentifications could have occurred due to the close relationship and overlapping characters of the sibling species within the Hyrcanus Group. Previous studies on *An. peditaeniatus* in Thailand confirm the zoophilic biting behavior [22, 47]. It has also been demonstrated that some *Anopheles* species may shift from zoophilic to anthropophilic behavior when the number of livestock decreases [48]. Some extrinsic parameters, such as local rainfall, season, latitude, deforestation, food source scarcity, habitat destruction and the use of insecticides, which vary accordingly to locations, may also impact the host preference and their resting behavior [49–53]. Large land-use changes have occurred in Ubon Ratchathani with severe deforestation for rice cultivation and conversion from rice paddy to rubber plantations that could have possibly influenced mosquito bionomics [54]. The early biting peak of *An. peditaeniatus* during dusk hours is of concern because human populations are often found outdoors during these periods and are not protected by bednets.

The results of the present 2-year study showed the very high abundance of specimens of the *An. hyrcanus* group, largely dominated by *An. peditaeniatus*, during the rainy season (90.5%). From previous studies, the preferential breeding habitat of the *An. hyrcanus* group was rice fields [24], which are widespread in Ubon Ratchathani Province. It could be hypothesized that increased transplanting and maintaining water in rice fields during the rainy season could influence the density of mosquito populations.

Only one *An. sinensis* specimen was identified by PCR out of the 603 *An. hyrcanus* (*s.l*.) tested. This mosquito species is widespread in China and Korea, and is considered an important malaria vector transmitting *P. vivax* [10, 55, 56]. It has been previously proved that *An. sinensis* populations could exhibit different vectorial capacity depending on their geographic location [56]. In Thailand, *An*. *sinensis* plays a minor role in malaria transmission due to its low abundance and restricted spatial distribution although some studies reported that it could be experimentally infected with *P. vivax* [57, 58]. However, further investigations are needed in order to clarify the vectorial role of *An*. *sinensis* in Thailand. Furthermore, although relatively low number of the other members of the Hyrcanus Group was found in this study, the role of these species as disease vectors may need to be considered. The role in malaria transmission of *An. nitidus*, *An. nigerrimus* and *An. argyropus* has not been documented so far. *Anopheles nigerrimus* and *An. argyropus* were reported as potential vectors of *B. malayi* experimentally, while *An. nitidus* was found to be a refractory vector [27]. Exploration on the vectorial role of these species could be valuable for malaria control strategies and surveillance.

Out of the 467 specimens of *An. peditaeniatus* tested in various bioassays (deltamethrin (0.05%), permethrin (0.75%), DDT (4%), with or without synergists), approximately half (49%) were found resistant to at least one of these insecticides. The addition of the PBO (Piperonyl butoxide) synergist, but not DEF (S.S.S-tributyl phosphotritioate), seemed to restore susceptibility, indicating a potential role of oxidases as a detoxifying enzyme resistance mechanism [59]. PBO and DEF are widely used as synergists for insecticide treatments. PBO inhibits cytochrome-P450 monooxygenases (multi-function oxidases) that mediates resistance to all classes of insecticides, while DEF is an enzyme inhibitor of esterases [60]. The other tested species in the *An. hyrcanus* group were susceptible to all three insecticides, although only 27 specimens were tested. A previous study along the Thai-Myanmar border reported *An. hyrcanus* (*s.l*.) mortality of 33% and 48% to deltamethrin (0.05%) and permethrin (0.75%), respectively [61]. Two alternate mutations L1014S and L1014F at residue L1014 of the voltage-gated sodium channel (VGSC) are associated with knockdown resistance in insects including anophelines [62]. The L1014S knockdown resistance (*kdr*) mutation has also already been detected in *An. peditaeniatus* [61]. Cross-resistance to DDT and pyrethroids was also observed in *An. peditaeniatus* from southern Vietnam with high frequency of the L1014F *kdr* allele and low frequency of the L1014S *kdr* allele [63]. These findings indicate that this species is under high selection pressure, probably due to the use of pesticides for crop protection.

## Conclusions

In Ubon Ratchathani Province, an unprecedented malaria outbreak occurred in 2014 with nearly 7-fold increment in the total number of malaria cases reported in 2013 [33]. To understand such outbreaks and widely assess the malaria risk in Thailand, it is important to study the spatial distribution of potential vectors and their bionomic traits including vectorial capacity, biting/resting behaviors and preferential breeding sites. Knowledge on these parameters are a prerequisite for implementing the most appropriate and efficient vector control programme. The occurrence and spread of insecticide resistance in malaria vectors, even in secondary or suspected vectors, could potentially change the dynamics of disease transmission and impact the efficacy of vector control interventions. The vectorial role of members of the *An. hyrcanus* group, as well as other secondary and suspected vectors regarding malaria in the Ubon Ratchathani Province and nationwide, needs to be further investigated in order to provide essential information for guiding vector-borne disease monitoring and control campaigns.

## **Supplementary information**

**Additional file 1: Table S1.** Collection locations of the Hyrcanus Group associated environment in Ubon Ratchathani Province, Thailand.

## Data Availability

The datasets used and/or analyzed during the present study are available from the corresponding author upon reasonable request.

## Abbreviations

AS-PCR: allele-specific polymerase chain reaction
CBC: cow bait collections
*cox*1: cytochrome *c* oxidase subunit 1
*cox*2: cytochrome *c* oxidase subunit 2
CSP: circumsporozoite protein
DDT: dichlorodiphenyltrichloroethane
DEF: S.S.S-tributyl phosphotritioate
DNA: deoxyribonucleic acid
dNTP: deoxynucleotide-5′-triphosphate
EDTA: ethylenediaminetetraacetic acid
ELISA: enzyme-linked immunosorbent assay
HLC: human-landing catch
ITS2: internal transcribed spacer 2
*kdr*: knockdown resistance
*P*: probability value
PBO: piperonyl butoxide
PCR: polymerase chain reaction
pH: potential of hydrogen ion
rDNA: ribosomal deoxyribonucleic acid
SPSS: Statistical Package for the Social Sciences
*s.l*.: *sensu lato*
*s.s*.: *sensu stricto*
Tris-HCl: Tris hydrochloride
