# Supplementary material for: Species diversity and insecticide resistance within the Anopheles hyrcanus group in Ubon Ratchathani Province, Thailand
Source: Parasit Vectors. 2020 Oct 17;13:525. doi: 10.1186/s13071-020-04389-4 (PMC7568835; doi:10.1186/s13071-020-04389-4)
Supplement: Supplementary file 1 — Additional file 1: Table S1. Collection locations of the Hyrcanus Group associated environment in Ubon Ratchathani Province, Thailand. [file 13071_2020_4389_MOESM1_ESM.docx]

| **Collection location** | **Sub-district** | **District** | **Environment** |
| --- | --- | --- | --- |
|  |  |  |  |
|  |  |  |  |
|  |  |  |  |
| **Pakla Village** | Na Pho Klang | Khong Chiam | Dry dipterocarp forests, cashew nut fields, rice fields, and evergreen trees. |
| **Talong Village** | Huai Pai | Khong Chiam | Located along the Mekong River surrounded by dry dipterocarp forests, cashew nut fields, rice fields, and evergreen trees. |
| **Payaka Village** | Non Ko | Sirindhorn | Seasonal evergreen forests, rubber plantations, and vegetable fields |
| **Nongmek Village** | Huai Kha | Buntharik | Rubber plantations, and cassava and rice fields. A stream runs near the collection site |
| **Sanghom Village** | Huai Kha | Buntharik | Cassava fields and other field crops, Rubber plantation and National Park. Natural running streams run through the site. Close to buffalo enclosure. |
| **Rubber plantation** | Nachaluay | Nachaluay | Rubber plantation. Palm oil plantations and a small dam are located nearby |

**Additional file 1: Table S1.** Collection locations of the Hyrcanus Group associated environment in Ubon Ratchathani Province, Thailand.
